# Prevalence and associated risk factors of resistant hypertension among Chinese hypertensive patients in primary care setting

**DOI:** 10.1186/s12875-024-02366-9

**Published:** 2024-04-19

**Authors:** Kilpatrick Kiupak Chan, Lapkin Chiang, Clarence Chuenming Choi, Yimchu Li, Catherine Xiarui Chen

**Affiliations:** 1 Department of Family Medicine and General Outpatient Clinics, Kowloon Central Cluster, Hospital Authority, Kowloon, Hong Kong SAR China; 2 Department of Family Medicine and General Outpatient Clinics, Queen Elizabeth Hospital, 30 Gascoigne Road, Kowloon, HK SAR China

**Keywords:** Resistant hypertension, Prevalence, Risk factors, Primary care

## Abstract

**Introduction:**

Hypertension (HT) is a major public health problem globally, and it is the commonest chronic disease with a prevalence of 27% among people aged 15 years or above in Hong Kong. There is emerging literature confirmed that patients with resistant hypertension (RHT) give its increased risk for adverse clinical outcomes and higher rate of documented target organ damage. This study aims to identify the prevalence of RHT among Chinese hypertensive patients managed in public primary care setting of Hong Kong and exploring its associated risk factors.

**Methodology:**

This is a cross-sectional descriptive study. Chinese hypertensive patients aged 30 or above with regular follow-up between 1st July 2019 and 30th June 2020 in 10 public primary care clinics under the Hospital Authority of Hong Kong were included. Demographic data, clinical parameters and drug profile of patients were retrieved from its computerized record system. The prevalence of RHT was identified and the associated risk factors of RHT were explored by multivariate logistic regression analysis.

**Results:**

Among the 538 sampled Chinese hypertensive patients, the mean age was 67.4 ± 11.5 years old, and 51.9% were female. The mean duration of hypertension was 10.1 ± 6.4 years, with a mean systolic and diastolic blood pressure of 128.8 ± 12.3 and 72.9 ± 10.8 mmHg respectively. 40 out of 538 patients were found to have RHT, giving an overall prevalence of 7.43%. Four factors were found to be associated with increased risk of RHT, in ascending order of odds ratio: duration of hypertension (OR 1.08), male gender (OR 2.72), comorbid with type 2 diabetes mellitus (T2DM, OR 2.99), and congestive heart failure (CHF, OR 5.39).

**Conclusion:**

The prevalence of RHT among Chinese hypertensive patients in primary care setting of Hong Kong is 7.43%. RHT is more common in male patients, patients with longer duration of hypertension, concomitant T2DM and CHF. Clinicians should be vigilant when managing these groups of patients and provide aggressive treatment and close monitoring.

## Introduction

Hypertension (HT) is a major public health problem with a global prevalence of 20–40% [1, 2]. In Hong Kong (HK), it is the commonest chronic disease with a prevalence of 27% among people aged 15 years or above [3], and the second commonest reason for consultation in primary care [4]. The “rule of halves” has been used to describe the situation in the management of HT that the rates of diagnosis, treatment, and control are all approximately 50% [3, 5].

Uncontrolled HT remains one of the most important cardiovascular risk factors in the world for myocardial infarction, heart failure, renal failure and cerebrovascular accidents [6, 7]. Resistant Hypertension (RHT) is defined as failure to achieve goal blood pressure (BP), i.e. <140/90 mmHg for overall population and < 130/80 mmHg for patients with diabetes mellitus (DM) or chronic kidney disease (CKD) despite adhering to maximally tolerated doses of three concurrent anti-hypertensive drugs including a diuretic, or requiring four or more concurrent anti-hypertensive drugs [8–10]. Despite a uniform definition now adopted by the US Joint National Committee [8], National Institute of Health and Clinical Excellence (NICE) [9] and the European Society of Cardiology [10], it is still challenging to estimate the actual prevalence of RHT, for apparent but false RHT due to suboptimal drug adherence [11] and the presence of white coat effect [12, 13] commonly exist in clinical practice. These are plausible explanations for the highly variable prevalence of RHT among studies, ranging from 6.5% in population-based cohorts study in the United Kingdom in 2015 [14], 7.9% and 8.8% respectively in cross-sectional studies nationwide in Korea [15] and regionally in Malaysia [16], 16.9% in Spanish ambulatory blood pressure monitoring registry [13], to 17% derived from Swedish primary care cardiovascular database [17].

There is emerging literature analysing the characteristics of patients with RHT given its increased risk for adverse clinical outcomes. When compared with those with white coat resistance, patients with true RHT were younger, more frequently men, smokers, diabetics, and had a longer duration of HT [12]. They had a higher rate of documented target organ damage including left ventricular hypertrophy, microalbuminuria, and a worse cardiovascular risk profile. High prevalence of diabetes, hyperlipidemia and target organ damage including heart failure, ischemic heart disease and CKD were seen in patients with RHT compared to patients without [17, 18].

A large proportion of Chinese patients with essential HT are managed in public primary care clinics of the Hospital Authority of HK (HAHK). While there is emerging literature analysing the characteristics of patients with RHT given its increased risk for adverse clinical outcomes, most of them were performed in the specialist setting and data from primary care is lacking. This study aims to fill this knowledge gap by identifying the prevalence of RHT among Chinese hypertensive patients managed in public primary care setting of HK and exploring its associated risk factors. We believe that early recognition of RHT, identification of its associated risk factors followed by aggressive treatment will help reduce the cardiovascular morbidity and mortality of all HT patients in the long run.

## Methods

### Study design

It is a cross-sectional descriptive study.

### Subjects

There are a total of 7 clusters in HAHK, with Kowloon Central Cluster (KCC) being the largest hospital cluster with a catchment of 1.2 million populations in 2019. Around 200,000 HT patients have regular follow-up (FU) in 10 public primary care clinics in KCC. The diagnosis of HT is based on the recommendations from NICE guideline [9]. These HT patients have FU every 1–6 months depending on their clinical condition. At least annually they receive blood tests including fasting sugar, lipid and renal function, and urine test for albumin.

In this study, Chinese HT patients age 30 or above with regular FU in these clinics between 1st July 2019 and 30th June 2020 inclusive were included. Age 30 was selected as cut-off since all young onset HT patients below age 30 are referred to specialist to rule out secondary causes.

HT patients with the following characteristics were excluded: (1) misdiagnosed HT such as white coat HT cases; (2) regular FU of HT in specialist out-patient clinics; (3) HT with a secondary cause; (4) ≤1 attendance for FU within the study period; (5) no documented BP within the study period; (6) no annual blood and urine tests done within the study period.

### Definition of resistant HT (RHT) and outcome measures

RHT is defined as (1) failure to achieve goal BP of < 140/90 mmHg for overall population and < 130/80 mmHg for patients with DM or CKD despite adhering to maximally tolerated doses of three concurrent antihypertensive drugs preferably including a diuretic, or (2) requiring four or more concurrent antihypertensive drugs use [8–10]. After ruling out the white coat effect, and ensuring drug adherence as documented in the case notes, patients fulfilling the definition of RHT were identified. The primary outcome is the prevalence of RHT among Chinese hypertensive patients managed in public primary care setting, and the secondary outcome is its associated risk factors from patients’ demographics and clinical parameters.

### Sample size calculation

The formula $n = \frac{{Z_{\alpha /2}^2P\left( {1 - P} \right)}}{{{E^2}}}$ was used to calculate the adequate sample size, where *n* = required minimum sample size, α = probability of type I error and Z_α_ = statistic corresponding to level of confidence, *P* = expected prevalence obtained from previous studies, and E = level of absolute precision. The estimated prevalence of RHT was used for sample size calculation since it is the primary outcome of this study. According to the literature, the prevalence of resistant HT in the community ranges from 6.5 to 17% [4–6]. Using α = 0.05 with Z_α_ = 1.96, *P* = 9.5%, E = 2.5%, the minimum sample size needed is 528. To allow room for case exclusion, 25% more patients up to 660 is needed for this study.

### Data Collection

The list of patients fulfilling the inclusion criteria was retrieved from the Clinical Data Analysis and Reporting System (CDARS) of HAHK. The list of hypertension patients were identified by code K86 (uncomplicated hypertension) and code K87 (complicated hypertension) based on Hong Kong Clinical Terminology Table (HKCTT). The list was then sequenced by their registration numbers, from which the sampled cases were selected based on computer-generated random numbers. The medical records of sampled patients were reviewed by the research team and their relevant data were captured from the Clinical Management System (CMS) of HAHK.

The latest clinic BP reading and antihypertensive regimen of every sampled patient were captured. Clinic BP is measured by self-service automated BP machines used in these primary care clinics, where two consecutive measurements are taken at least 1 min apart with the patient seated. If one of the readings is ≥ 140/90 mmHg, a third measurement is taken during the consultation. The lower of the latter two measurements would be recorded as the clinic BP [9]. Home BP readings if charted, ambulatory BP readings if performed, and white coat effect if present, were also documented. Antihypertensive drugs are classified into: renin-angiotensin-system inhibitors comprising angiotensin-converting enzyme inhibitors (ACEI) and angiotensin receptor blockers (ARB), beta-blockers (BB), calcium channel blockers (CCB), diuretics, alpha-blockers, central-acting agents and vasodilators. If any of these drugs were not prescribed for BP control but solely for other indications, such as ACEI or ARB for controlling microalbuminuria and proteinuria, and alpha-blockers for relieving lower urinary tract symptoms, they would not be counted in the antihypertensive drug regimen.

Demographic data included age, gender, smoking and drinking status, and clinical characteristics included body mass index (BMI) and duration of HT were recorded. BMI is calculated as [body weight / height^2^] in kg/m^2^. Duration of HT is rounded off to the nearest 0.5 years after its diagnosis as recorded in the CMS. Levels of serum fasting sugar, lipid profile, creatinine and urine albumin, and electrocardiogram findings if done, were retrieved from CMS. The latest results were recorded if tests had been performed more than once within the study period. The Modification of Diet in Renal Disease Study equation [eGFR = 186×(SCR×0.011)-1.154×(age)-0.203 × (0.742 [if female])×1.233 where SCR = serum creatinine expressed in µmol/L] was used to estimate the glomerular filtration rate (eGFR), expressed in mL/min/1.73 m^2^ (www.kidney.org/content/mdrd-study-equation). CKD is defined as eGFR < 60 ml/min/1.73m^2^. Comorbidities including DM, hyperlipidemia, stroke, ischemic heart disease (IHD), left ventricular hypertrophy (LVH), congestive heart failure (CHF), atrial fibrillation (AF), peripheral vascular disease (PVD), gout, obstructive sleep apnoea (OSA) and benign prostatic hypertrophy (BPH) were identified by reviewing patients’ medical records in the CMS.

### Statistical analysis

All statistical analyses were performed using the Statistical Package for Social Sciences (SPSS version 23). Continuous data are described as mean and standard deviation if the distribution is normal. Categorical data are reported as percentages. For univariate analyses, Student’s t test was used for continuous variables, and Chi-square or Fisher exact tests (if the number of the categorical data ≤5) were used for categorical variables. Independent variables with *p* < 0.1 in the univariate analyses were entered into a multivariate logistic regression analysis to identify associated risk factors of RHT expressed as odds ratio (OR). All analyses were performed with 95% confidence intervals (95% CI) and level of significance as *p* < 0.05.

## Results

**Fig. 1:**
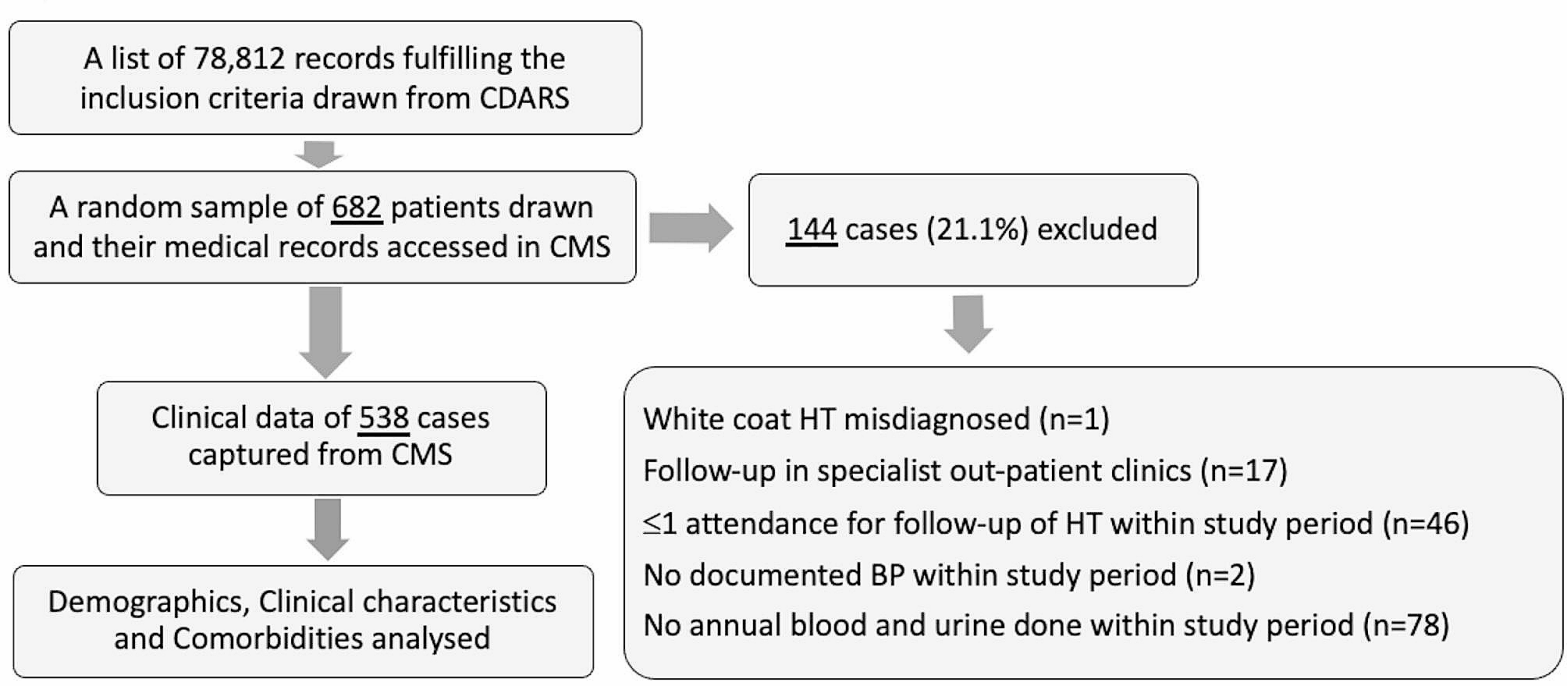
Flowchart of study

A total of 78,812 patients fulfilling the inclusion criteria were identified from the CDARS of HAHK during the study period. Among the randomly sampled 682 HT patients, 144 (21.1%) were excluded due to the following reasons: 1 due to misdiagnosed HT, 17 due to FU in specialist out-patient clinics, 46 due to ≤1 attendance during the study period, 2 due to no documented BP and 78 due to no annual blood and urine done within the study period. No case of HT with a secondary cause was identified. Clinical data of the remaining 538 cases were collected from the CMS and entered into data analysis (Fig. 1).

Table 1 summarized the demographics and characteristics of all included HT patients. Their mean age was 67.4 ± 11.5 years, and 51.9% were female. 14.3% of patients were current smoker, 5.6% were current drinker, while 57.6% were obese. Majority of patients had associated comorbidities, including 40.1% had DM, 80.1% had hyperlipidaemia, 15.4% had CKD, 12.6% had stroke and 10.2% had IHD. The mean duration of HT was 10.1 ± 6.4 years, and the mean systolic and diastolic BP were 128 ± 12.3 and 74 ± 10.8 mmHg respectively. Among them, 40 cases were identified to have RHT, giving a prevalence of 7.43%.

**Table 1:** Demographics and characteristics of total patients, patients with and without RHT

	Total (*n* = 538)	Non-RTH (*n* = 498)	RTH (*n* = 40)	Non-RHT vs. RHT
	Number (% out of Total/Non-RHT/RHT) or #Mean ± SD			*P*-value
**Demographics**				
Age (years)	67.4 ± 11.5^#^	67.4 ± 11.6^#^	69.9 ± 10.5^#^	0.820
Female	279 (51.9)	266 (53.4)	13 (32.5)	-
Male	259 (48.1)	232 (46.6)	27 (67.5)	0.011
Current smoker	77 (14.3)	72 (14.5)	5 (12.5)	0.734
Current drinker	30 (5.6)	26 (5.2)	4 (10.0)	0.268
**Clinical parameters**				
Body Mass Index (BMI, kg/m^2^) Underweight (< 18.5) Normal (18.5–22.9) Overweight (23–24.9) Obesity (>/=25)	11 (2.1) 114 (21.2) 103 (19.1) 310 (57.6)	10 (2.0) 110 (22.1) 96 (19.3) 282 (56.6)	1 (2.5) 4 (10.0) 7 (17.5) 28 (70.0)	0.283 - - - -
Duration of hypertension (years)	10.1 ± 6.4^#^	9.8 ± 6.2^#^	13.7 ± 7.4^#^	< 0.001
Number of antihypertensive agents One Two Three Four Five	243 (45.2) 194 (36.1) 67 (12.4) 30 (5.6) 4 (0.7)	243 (48.8) 194 (39.0) 60 (12.0) 1 (0.2) 0	0 0 7 (17.5) 29 (72.5) 4 (10.0)	- - - - -
Systolic blood pressure (mmHg)	128.8 ± 12.3^#^	128.2 ± 11.7^#^	136.3 ± 16.2^#^	0.003
Diastolic blood pressure (mmHg)	72.9 ± 10.8^#^	72.8 ± 10.5^#^	74.8 ± 14.0^#^	0.374
**Resistant Hypertension (RHT)**	40 (7.43)	0 (0.0%)	40 (100%)	-
**Comorbidities**				
Diabetes mellitus	216 (40.1)	190 (38.2)	26 (65.0)	0.001
Hyperlipidaemia	431 (80.1)	397 (79.7)	34 (85.0)	0.434
Chronic kidney disease	83 (15.4)	70 (14.1)	13 (32.5)	0.002
Microalbuminuria	86 (16.0)	74 (14.9)	12 (30.0)	0.012
Stroke	68 (12.6)	59 (11.8)	9 (22.5)	0.051
Ischaemia heart disease	55 (10.2)	48 (9.6)	7 (17.5)	0.167
Left ventricular hypertrophy	29 (5.4)	24 (4.8)	5 (12.5)	0.055
Congestive heart failure	12 (2.2)	8 (1.6)	4 (10.0)	0.008
Atrial fibrillation	21 (3.9)	19 (3.8)	2 (5.0)	0.664
Peripheral vascular disease	1 (0.2)	1 (0.2)	0 (0.0)	1.000
Gout	34 (6.3)	28 (5.6)	6 (15.0)	0.032
Obstructive sleep apnea	27 (5.0)	25 (5.6)	2 (5.0)	1.000
Benign prostatic hypertrophy	66 (25.5, male = 259)	61 (26.3, male = 232)	5 (18.5, male = 27)	0.380

Table 1 also compared the demographic and clinical parameters in patients with and without RHT. There were statistically more male patients (67.5% versus 32.5%) in the RHT group (*p* = 0.011). RHT patients were found to have longer duration of HT than non-RHT patients (13.7 ± 7.4 years versus 9.8 ± 6.2 years, *p* = 0.0002). Patients with RHT had statistically higher proportions of comorbidities: DM (65.0% versus 38.2%, *p* = 0.001); CKD (32.5% versus 14.1%, *p* = 0.002); microalbuminuria (30.0% versus 14.9%, *p* = 0.012); CHF (10.0% versus 1.6%, *p* = 0.008); and gout (15.0% versus 5.6%, *p* = 0.032).

In addition to the seven statistically significant variables associated with RHT, stroke and LVH also had *p* < 0.1 and were hence entered into multivariate logistic regression to identify associated risk factors of RHT, as shown in Table 2. Adjusting all nine variables forward stepwise in the model, increased risk of RHT were statistically associated with four factors, in ascending order of odds ratio: duration of HT (OR 1.08, 95%CI 1.03–1.14), male gender (OR 2.72, 95%CI 1.33–5.56), comorbid with type 2 DM (T2DM) (OR 2.99, 95%CI 1.49-6.00), and CHF (OR 5.39, 95%CI 1.41–20.69).

**Table 2:** Associated risk factors of resistant hypertension

	Adjusted OR	95% C.I.	*p*-value
Male gender	2.72	1.33–5.56	0.005
Duration of hypertension	1.08	1.03–1.14	0.001
Diabetes mellitus	2.99	1.49–6.00	0.002
Chronic kidney disease	1.56	0.68–3.61	0.297
Microalbuminuria	1.04	0.45–2.40	0.924
Stroke	1.38	0.56–3.40	0.481
Left ventricular hypertrophy	2.24	0.71–7.05	0.168
Congestive heart failure	5.39	1.41–20.69	0.014
Gout	1.34	0.45–4.00	0.595

**Fig. 2:**
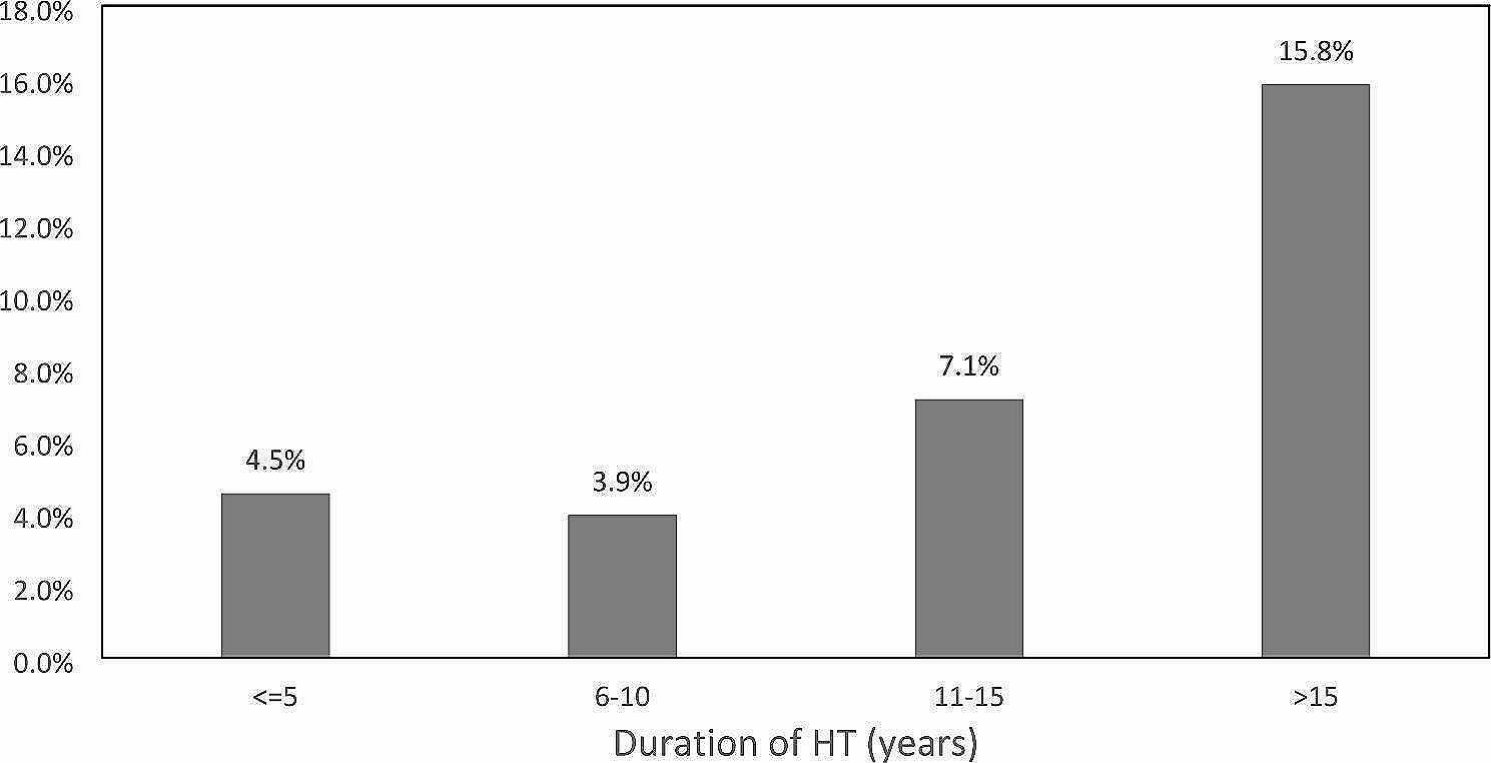
Proportion of RHT among HT patients of different durations

The duration of HT as an associated risk factor was further analysed as shown in Fig. 2. Patients with HT over 10 years were nearly twice more likely to have RHT compared to those diagnosed within 10 years, and nearly four times more likely when the duration is over 15 years (*p* = 0.0007).

**Fig. 3:**
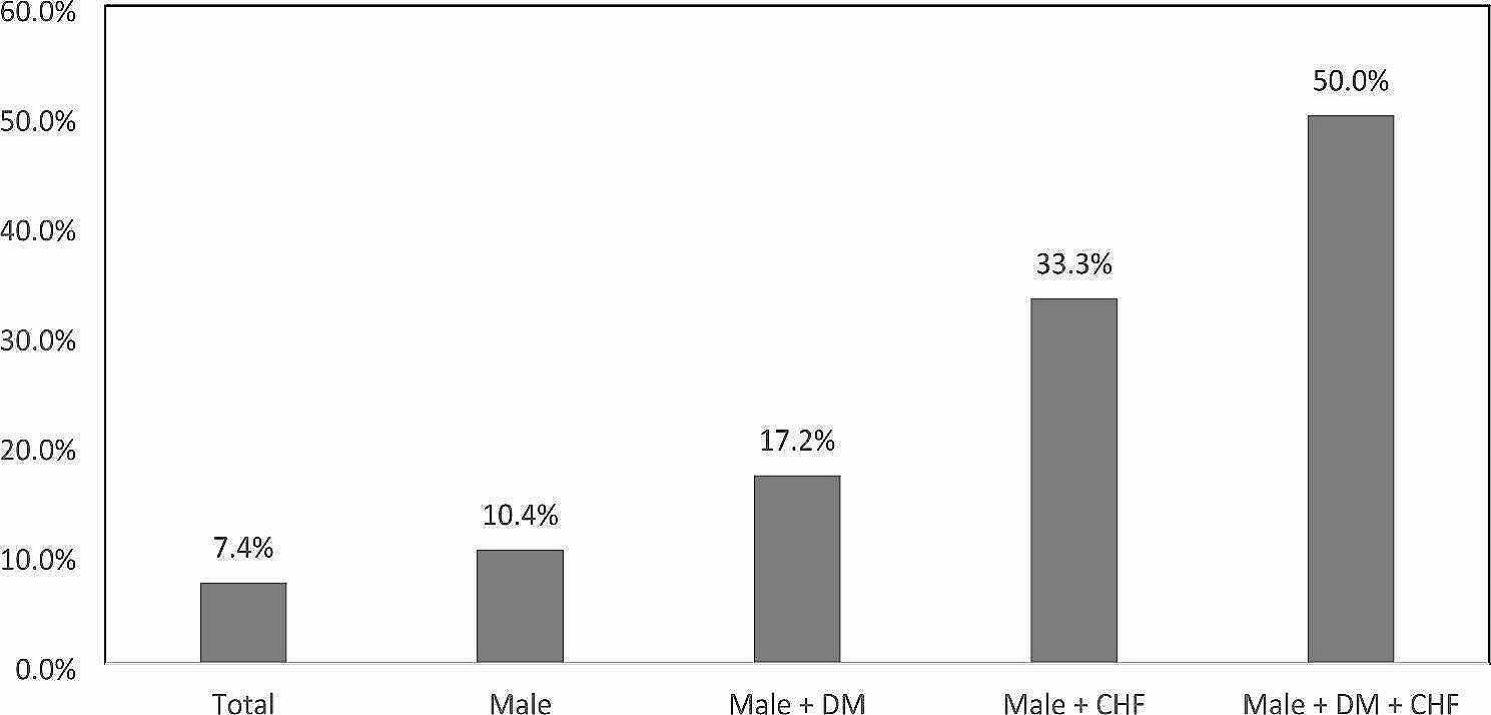
Proportion of RHT among patients with different associated risk factors

Figure 3 demonstrates the interactions among the other three risk factors associated with RHT. One in ten male HT patients had RHT and this ratio increased to one in six if they were also diabetic. One-third of male HT patients with CHF had RHT, and the risk increased to one in half if the male HT patients had both T2DM and CHF concomitantly (*p* = 0.003).

## Discussion

Our study found that 7.43% of Chinese hypertensive patients from primary care have RHT. This figure is comparable with those reported from similar studies done in Korea, Malaysia, and the United Kingdom [14–16], with a prevalence of RHT ranging from 6.5 to 8.8%. Not surprisingly, these figures are much lower than that 14% reported from a study conducted in a secondary care setting [19]. This discrepancy can be explained by a higher proportion of secondary HT as well as advanced diseases in the specialist setting. In addition, HT patients with multi-comorbidities including T2DM, CKD and CHF, are usually managed in the secondary care, where more antihypertensive drugs are needed to bring their BP to target.

Higher proportion of associated target organ damage were found in RHT patients compared with non-RHT patients in our study. Significantly higher comorbid rates of CKD, microalbuminuria and CHF were noted in the RHT group. The comorbid rates of stroke, IHD and LVH were also higher in the RHT group though the difference was not statistically significant. These findings were similar to those from the Swedish primary care cardiovascular database showing higher frequencies of CKD, CHF and IHD in RHT patients compared to those without [17], and higher prevalence of IHD (43%) and LVH (40%) observed in RHT patients in another population-based study in Italy [18]. Complicated HT patients are more refractory to treatment, whereas RHT patients are more prone to develop severe complications, and this vicious cycle indeed underpins the importance of identifying RHT cases early in our daily practice. In this regard, a broader preventive approach to actively screen for target organ damage among HT patients, as well as an aggressive BP control to prevent the development of HT related complications should be promoted, particularly in primary care.

As to the associated risk factors of RHT, our study showed that RHT was more common in hypertensive patients whom were male and had longer duration of HT, which was in line with a large population-based cohort study with exactly the same observation [12]. In particular, our study revealed that having HT for over 10 years will render the patient two times more likely to be resistant to treatment, and the prevalence will reach four times when the duration is over 15 years. Given the aging population both in HK and worldwide, as well as the finding that the incidence of hypertension is rising faster in young adults than in older adults [20], HT patients are more likely to endure a longer duration of HT over lifetime, thus rendering them an increased risk of developing RHT in the future.

Our study also demonstrated strong association between T2DM and CHF with RHT, which were consistent with findings from the emerging evidence [12, 17, 18]. This is partially due to the fact that multiple anti-hypertensives are often required to achieve a more stringent BP target of < 130/80 mmHg for T2DM patients. In addition, the presence of insulin resistance and a higher rate of metabolic syndrome in T2DM patients contribute to their being refractory to antihypertensive treatment [21]. Furthermore, being comorbid with CHF was found to have the strongest association with RHT in our study. Indeed, CHF had been reported to be the most common comorbidity among RHT patients from the Swedish primary care cardiovascular database [17]. As HT patients with CHF are often put on BB and diuretics as a compelling indication for improving their heart function and alleviating ankle oedema, in addition to the first-line anti-hypertensives such as ACEI or ARB or CCB [9], it is not surprising that HT patients with CHF may fulfil the definition of RHT more easily. It is alarming to find that the prevalence of RHT would reach 50%, which is seven times higher than that of the overall HT population, if all three risk factors including male gender, comorbid with DM and CHF are present. Given that RHT leads to an increased all-cause mortality and cardiovascular outcomes, special attention should be paid to this high risk group of patients and a more proactive approach with closer monitoring is needed to bring their BP to target.

This is the first study to investigate the prevalence of RHT in primary care setting in HK and has provided important background information on this topic and its associated risk factors. All the clinical data, biochemical measurements and drug profile were retrieved from the computerized system in HA, thus minimizing risk from recall bias or manual errors. That being said, there are several limitations in this study. First, the study was carried out in one single cluster of HA and therefore selection bias might exist. These results from the public primary health care sector might not be applicable to the private sector or secondary care. Second, 78 HT cases (11.4% of the sampled cases) were found to have no annual blood or urine check-up during the study period and were excluded from the data analysis, which might affect the accuracy of the study outcome. Third, owing to the cross-sectional nature of the study, we were unable to adjust for potential unmeasured confounders and hence no temporal or causal relationship could be established.

## Conclusion

7.43% of Chinese hypertensive patients managed in primary care were found to have RHT. It is more common in male hypertensive patients and patients with longer duration of HT, and is strongly associated with comorbid T2DM and CHF. Clinicians should be vigilant when managing these groups of patients and take a more proactive approach with closer monitoring as appropriate so as to improve their clinical outcome in the long run.

## Data Availability

The datasets generated and/or analysed during the current study are not publicly available to protect the confidentiality of participants’ data but are available from the corresponding author upon reasonable request,
